# Notes on the ant genus *Cataglyphis* Foerster, 1850 (Hymenoptera, Formicidae) in the Arabian Peninsula with description of a new species and a key to species of the *C. pallida*-group

**DOI:** 10.3897/zookeys.545.6308

**Published:** 2015-12-14

**Authors:** Mostafa R. Sharaf, Cedric A. Collingwood, Abdulrahman S. Aldawood

**Affiliations:** 1 Economic Entomology Research Unit (EERU), Plant Protection Department, College of Food and Agriculture Sciences, King Saud University, Riyadh 11451, P. O. Box 2460, Saudi Arabia; 2 18 Milton Street, Skipton, North Yorkshire, BD23 2ED, U. K.

**Keywords:** Synonymy, new designation, taxonomy, lectotype, Arabian Peninsula, Middle East, Palearctic region, Formicinae, key

## Abstract

*Cataglyphis fisheri* **sp. n.** is described and illustrated from the United Arab Emirates, Oman and Kingdom of Saudi Arabia based on the worker caste. It belongs to the *Cataglyphis pallida*-group which is recorded for the first time from the Arabian Peninsula. *Cataglyphis fisheri* **sp. n.** is similar to *Cataglyphis pallida* Mayr, 1877 from Kazakhstan. Differential diagnosis between these two species is given and a key to the species of the *Cataglyphis pallida*-group is presented. *Cataglyphis laylae* Collingwood, 2011 is treated as a junior synonym of *Cataglyphis saharae* Santschi, 1929. *Cataglyphis flavobrunnea* Collingwood & Agosti, 1996 is redescribed and a lectotype for this species is designated.

## Introduction

The ant genus *Cataglyphis* Foerster, 1850 currently includes 108 valid species and subspecies (http://www.antwiki.org/, accessed 15 August 2015) distributed in the desert areas of the Palearctic Region ranging from South Palearctic to Ghana, East to North China and India (Brown 2000). Members of the genus are among the commonest ants of the desert ecosystems of the Arabian Peninsula and Central Asia, where they build their crater nests directly in the ground (Brown 2000, Sharaf and Aldawood, unpublished data) and feed on dead insects (Lenoir et al. 2010). Their role in the desert ecosystem is important as they have been reported pollinating flowering plants (Herrera et al. 1984) and contributing to the dispersal of seeds (Hulme 1997; Boulay et al. 2007).

The worldwide revision of the genus by Santschi (1929) is out of date, but a comprehensive reclassification of the genus and its species groups was presented by Agosti in 1990. Many regional faunal treatments are available: Israel (Emery 1925), Iraq (Pisarski 1965), former European U.S.S.R. (Arnol’di and Dlussky 1978), Iberian Peninsula (Collingwood 1978), Kingdom of Saudi Arabia (KSA) (Collingwood 1985), Turkmenistan (Dlussky, Soyunov and Zabelin 1992), Bulgaria (Atanasov and Dlussky 1992), Armenia (Arakelian 1994), Central Europe (Seifert 1996), Portugal (Collingwood and Prince 1998), Asia species key (Radchenko 1998), Northwest China (Chang and He 2002), Egypt (Sharaf 2006), North and Central Europe species key (Seifert 2007) and Morocco (Cagniant 2009). Many *Cataglyphis* species are polymorphic, dimorphic or have variation in worker size. This makes it necessary to be cautious when making identifications and, even more, so when treating a single worker as representing a new species.

The Arabian species of *Cataglyphis* were first treated by Collingwood (1985), who recorded 18 species from the KSA describing two new species *Cataglyphis asiriensis* and *Cataglyphis minima* from the Asir Mountains of southwestern KSA. Subsequently, Collingwood and Agosti (1996) reviewed the genus for the entire Arabian Peninsula providing a key to species. The 26 recorded species included six new species, *Cataglyphis acutinodis*, *Cataglyphis flavobrunnea*, *Cataglyphis harteni*, *Cataglyphis holgerseni*, *Cataglyphis opacior* and *Cataglyphis shuaibensis*. A myrmecofaunal list of the United Arab Emirates (UAE) (Collingwood et al. 2011) reported 20 species and described an additional new species, *Cataglyphis laylae* Collingwood for the Peninsula.

In the present paper a new species, *Cataglyphis fisheri*, is described from the UAE based on the worker caste. A new lectotype designation for *Cataglyphis flavobrunnea* Collingwood & Agosti, 1996 is presented with redescription of the worker caste. *Cataglyphis laylae* Collingwood, 2011 is shown to be a junior synonym of *Cataglyphis saharae* Santschi, 1929.

## Materials and methods

### Measurements and indices

All measurements are in millimeters and follow standard measurements of Agosti (1990).

**Measurements**

**Eye length (EL)** Maximum diameter of eye.

(F1) Length of first funicular segment.

(F2) Length of second funicular segment.

**Head length (HL)** Length of head proper, excluding mandibles, measured from mid-point of anterior clypeal margin to mid-point of posterior head margin, in full-face view.

**Head width (HW)** Maximum width of head in full-face view, measured below eyes.

**Metanotum height (MH)** Maximum distance from line spanned between anteriormost and posteriormost part of mesosoma and lowest part of metanotum, measured at a right angle to this line.

**Mesosomal length (ML)** Diagonal length of mesosoma in profile from point at which pronotum meets the cervical shield to posterior base of metapleuron.

**Median ocellus size (OS)** Diameter of the ocelli.

**Ocelli distance (OD)** Distance between the two basal ocellus.

**Propodeum height (PH)** Maximum distance from a line spanned between anteriormost and posteriormost part of mesosoma and most raised part of propodeum, measured at a right angle to this line.

**Pronotal width (PW)** Maximum width of pronotum measured in dorsal view.

**Petiole height (PTH)** Maximum height of petiolar measured in lateral view from highest (median) point of node to ventral outline.

**Scape length (SL)** Maximum straight line length of antennal scape excluding basal constriction or neck to condylar bulb.

**Total length (TL)** Outstretched body length from mandibular apex to gastral apex.

**Indices**

**Cephalic index (CI)** HW × 100/HL.

**Eye index (EI)** EL × 100/HW.

**Funicular index (FI)** Length of first funicular segment × 100/Length of second funicular segment.

**Propodeum index (PI)** PH × 100/MH.

**Scape index (SI)** SL × 100/HW.

### Abbreviations of depositories

KSMA King Saud University Museum of Arthropods, King Saud University, College of Food and Agriculture Sciences, Plant Protection department, Riyadh, Kingdom of Saudi Arabia.

MHNG Muséum d’Histoire Naturelle de la Ville de Genève, Geneva, Switzerland.

NHMB Naturhistorisches Museum Basel, Basel, Switzerland.

WMLC World Museum Liverpool, Liverpool, United Kingdom.

In the original description of *Cataglyphis flavobrunnea* Collingwood and Agosti fixed the holotype from Oman and listed nine paratype specimens from Oman, The KSA, The United Arab Emirates and Yemen. Extensive searches in WMLC and NHMB did not succeed in finding the holotype specimen but nine specimens matching the paratypes data from the KSA were located. As recommended by the International Commission of Zoological Nomenclature, we designate a lecotype in this study to unequivocally ascertain the identity of the species

## Results and discussion

### Cataglyphis fisheri

Taxon classificationAnimaliaHymenopteraFormicidae

Sharaf & Aldawood sp. n.

http://zoobank.org/9C54C443-0729-42AE-9B3A-E7CA294879E9

Figures 1 , 2 , 3

#### Material examined.

United Arab Emirates, Baynounah, “sandy desert” (Sheiekh Zayed city), 23°38'40"N 53°37'12"E, 8.iii.1995, (C. A. Collingwood leg.), next to *Zygophyllum* plants, King Saud Museum of Arthropods (KSMA), College of Food and Agriculture Sciences, King Saud University, Riyadh, Kingdom of Saudi Arabia.

#### Paratypes.

1 worker, United Arab Emirates, Rhatam, 11.xi.1993, (C. A. Collingwood leg.), (KSMA); 3 workers, Oman desert, 30.ii.1997, (M. D. Gallagher leg.), (code 8907) (WMLC); 1 worker, Saudi Arabia, Riyadh Province, Rawdhat She’al, 22.40318°N, 46.59209°E, 596m, 13.iv.2015, PT (Aldhafer et al. leg.) (KSMA); 4 worker, Saudi Arabia, Riyadh Province, Rawdhat She’al, 22.41559°N, 46.58806°E, 602m, 18.x.2015, PT (Aldhafer et al. leg.) (KSMA); 7 worker, Saudi Arabia, Riyadh Province, Rawdhat She’al, 22.4279°N, 46.57547°E, 612m, 18.x.2015, PT (Aldhafer et al. leg.) (KSMA); 2 worker, Saudi Arabia, Riyadh Province, Rawdhat She’al, 22.42496°N, 46.57556°E, 606m, 18.x.2015, PT (Aldhafer et al. leg.) (KSMA).

#### Holotype worker.

TL 5.00, F1 0.26, F2 0.15, HL 1.24, HW 1.16, MH 0.20, PH 0.22, SL 1.27, ML 1.80, OS 0.07, OD 0.08, EL 0.28, PW 1.00, PTH 0.50, CI 94, EI 24, FI 173, PI 110, SI 109.

#### Paratype.

TL 3.12–5.75, F1 0.17–0.30, F2 0.10–0.17, HL 0.87–1.32, HW 0.72–1.17, MH 0.10–0.17, PH 0.15–0.25, SL 0.97–1.25, ML 1.37–2.00, OS 0.07, OD 0.07-0.08, EL 0.20–0.35, PW 0.55–0.95, PTH 0.22–0.42, CI 77–90, EI 25–34, FI 113–220, PI 125–208, SI 90–144 (11 measured).

#### Worker.

**Head.** Head distinctly longer than broad (CI 83–94), with straight posterior and lateral margins; posterior ocelli located at the level of posterior margin of eyes; scapes when laid back from their insertions surpass posterior margin of head by less than ¼ of its length. **Mesosoma.** Propodeal dorsum in profile distinctly low, nearly at same level as petiolar node. **Petiole.** Petiole an upright or slightly inclined scale, with the anterior face meeting the posterior face with a narrowly rounded margin angle. **Pilosity.** Third maxillary palp with erect hairs not longer than 1.5× maximum diameter of third segment; area behind the lateral clypeal margins with dense white pubescence, anterior clypeal margin with abundant long, curved, hairs; mesosomal dorsum with a few scattered hairs, two pairs each on the mesonotum and propodeum, petiole bare, gaster bare except for a few short hairs on apex, posterior margin of head with single pair of erect hairs. **Sculpture.** Cephalic dorsum faintly sculptured; median cephalic surface in front of ocelli feebly but distinctly longitudinally striated, striae curving outward to lateral margins in front of eyes; mandibles faintly but distinctly longitudinally striated, mesosoma and petiole faintly sculptured, general appearance dull. **Colour.** Uniform yellow, mandibular teeth brown.

**Figures 1.**
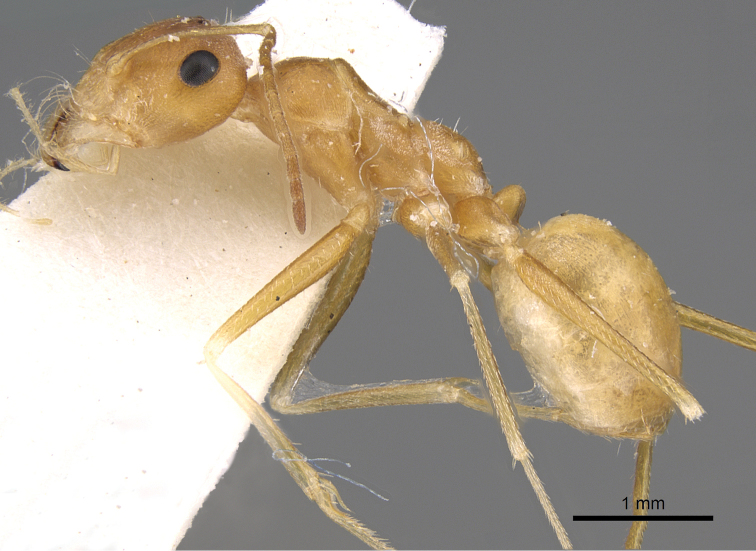
Body in profile of *Cataglyphis fisheri* sp. n. (Holotype worker), CASENT0906454. Photographer: Cerise Chen, www.AntWeb.org

**Figures 2.**
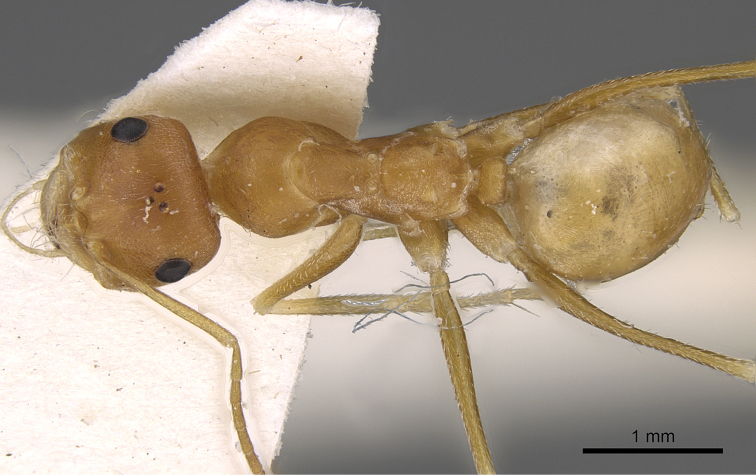
Body in dorsal view of *Cataglyphis fisheri* sp. n. (Holotype worker), CASENT0906454. Photographer: Cerise Chen, www.AntWeb.org

**Figures 3.**
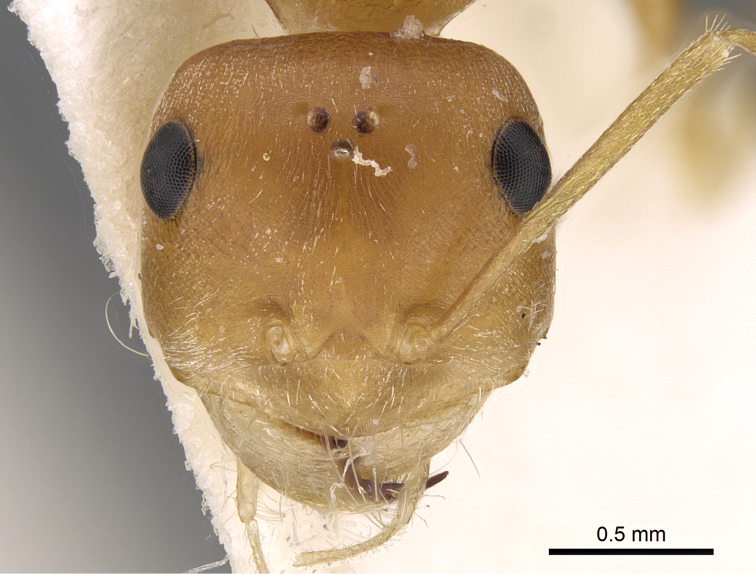
Head in full-face view of *Cataglyphis fisheri* sp. n. (Holotype worker), CASENT0906454. Photographer: Cerise Chen, www.AntWeb.org

#### Etymology.

This species is named in honor of Dr. Brian Fisher, Department of Entomology, California Academy of Sciences, San Francisco, California, U.S.A.

#### Affinities.

*Cataglyphis fisheri* is a member of the *Cataglyphis pallida*-group as defined by Agosti (1990), which is recorded here for the first time from the Arabian Peninsula. Although Collingwood and Agosti (1996) reported 265 species or morhospecies from the entire Arabian Peninsula, a large number of ant specimens remained unidentified. *Cataglyphis fisheri* was among those specimens. The workers cannot be identified from the key to Arabian species compiled by Collingwood and Agosti (1996) because material was not included in the study. However, *Cataglyphis fisheri* is similar to the Palearctic species *Cataglyphis pallida* Mayr, 1877 described from Kazakhstan. A differential diagnosis of the two species is summarized in Table 1.

**Table 1.** Differential diagnosis between *Cataglyphis fisheri* sp. n. and *Cataglyphis pallida*.

*Cataglyphis fisheri* sp. n.	*Cataglyphis pallida*
Posterior margin of head in full-face view straight and with five hairs.	Posterior margin of head in full-face view convex and without hairs.
Head and mesosoma with few pubescence.	Head and mesosoma with abundant pale pubescence.
Median cephalic surface in front of ocelli feebly but distinctly longitudinally striated, the striae curving outward to lateral margins in front of eyes.	Cephalic surface unsculptured.
Ocelli larger and set closer together	Ocelli smaller and set apart from each other.
OS 0.07, OD 0.07–0.08	OS 0.04, OD 0.10
Scape shorter, SL 0.97–1.25, SI 90–144	Scape longer, SL 1.30, SI 173

In the key to Arabian species (Collingwood and Agosti 1996), *Cataglyphis fisheri* will run to couplet 12 that also includes the much larger *Cataglyphis sabulosa* Kugler, 1981. *Cataglyphis fisheri* sp. n. can be readily separated by the upright or slightly inclined petiole with a dorsal rounded node, the lower propodeal profile and the absence of body pubescence whereas *Cataglyphis sabulosa* has a squaminode petiole with a convex anterior surface and straight posteriorly, a higher propodeal profile and the body covered with white silvery pubescence. The drawing of *Cataglyphis sabulosa* in Collingwood (1985) is incorrect.

### Key to species of *Cataglyphis pallida*-group

**Table:** 

1	Colour uniform brown or dark brown; mesosoma massively constructed; in profile propodeal dorsum high, meeting declivity in a distinct obtuse angle (Figure 4) Kazakhstan (type locality), Afghanistan, China, Iran, Turkmenistan	***emeryi* (Karavaiev, 1911)**
–	Colour uniform pale yellow or orange yellow; mesosoma elegantly or smoothly constructed; in profile propodeal dorsum making a continuous curve into the declivity (Figure 5)	**2**
2	Ocelli smaller and set apart from each other (OS 0.04, OD 0.10) (Figure 6); posterior margin of head in full-face view convex and without hairs (Figure 6); cephalic surface unsculptured (Figure 6); body colour pale yellow (Figure 7). Kazakhstan (type locality), Afghanistan, China, Kyrgyzstan, Turkmenistan	***pallida* Mayr, 1877**
–	Ocelli larger and set closer together (OS 0.07, OD 0.07–0.08); posterior margin of head in full-face view straight and with a single pair of hairs (Figure 8); median cephalic surface in front of ocelli feebly but distinctly longitudinally striated, the striae curving outward to lateral margins in front of eyes (Figure 8); body colour orange yellow. Kingom of Saudi Arabia	***fisheri* sp. n.**

**Figure 4.**
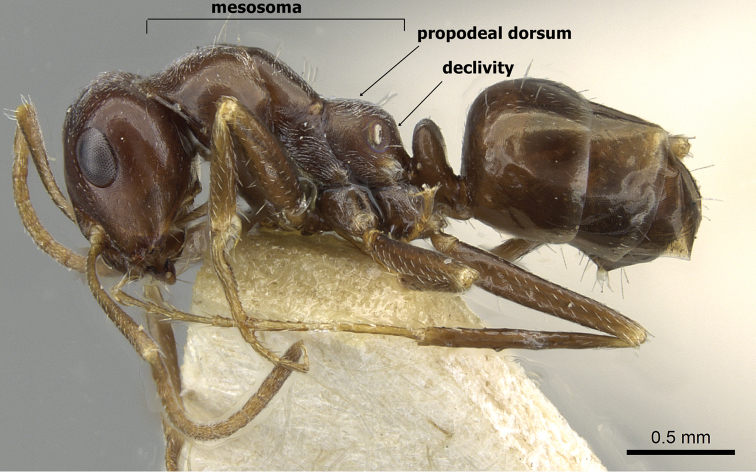
*Cataglyphis emeryi* body in profile, (Syntype worker), CASENT0911110, Photographer: Zach Lieberman.

**Figure 5.**
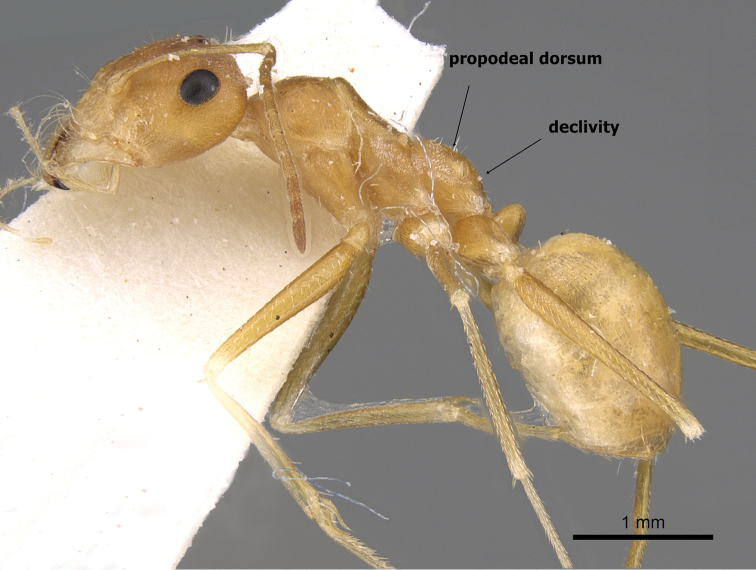
*Cataglyphis fisheri* sp. n. (Holotype worker), body in profile, CASENT0906454, Photographer: Cerise Chen.

**Figure 6.**
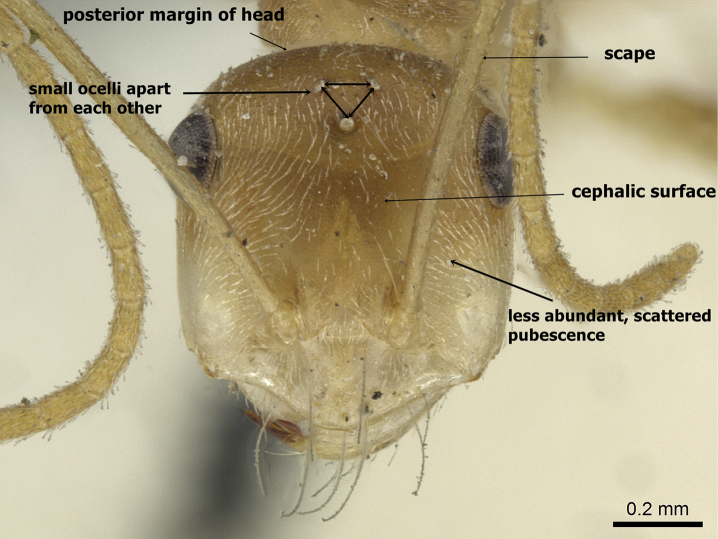
*Cataglyphis pallida*, head in full-face view, CASENT0911112, Photographer: Zach Lieberman.

**Figure 7.**
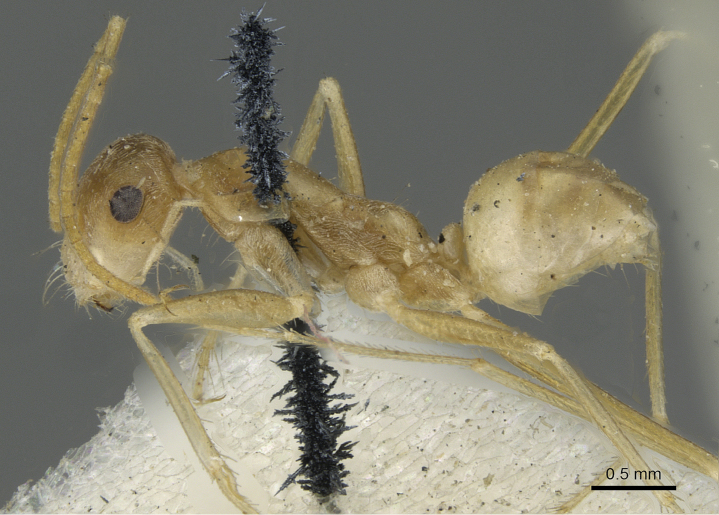
*Cataglyphis fisheri* sp. n. (Holotype worker), body in profile, CASENT0906454, Photographer: Cerise Chen.

**Figure 8.**
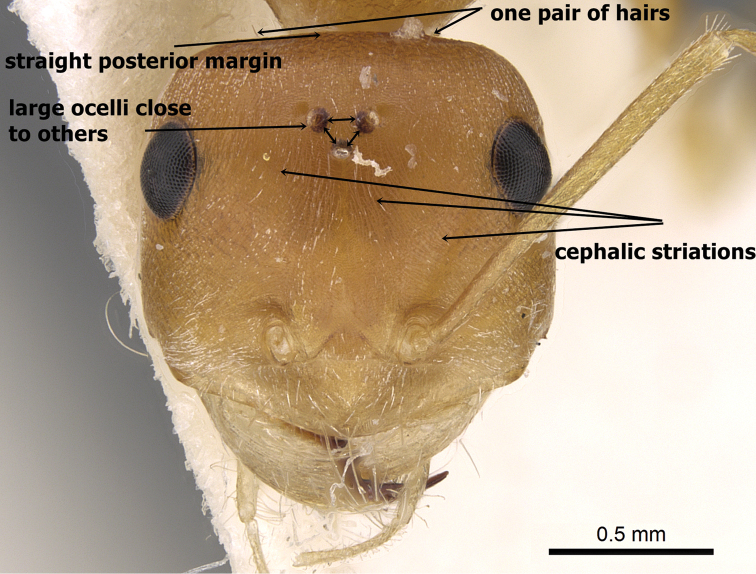
*Cataglyphis pallida* (Cotype), head in full-face view, CASENT0911112, Photographer: Alexandra Westrich.

### Cataglyphis flavobrunnea

Taxon classificationAnimaliaHymenopteraFormicidae

Collingwood & Agosti, 1996

**Figure 9.**
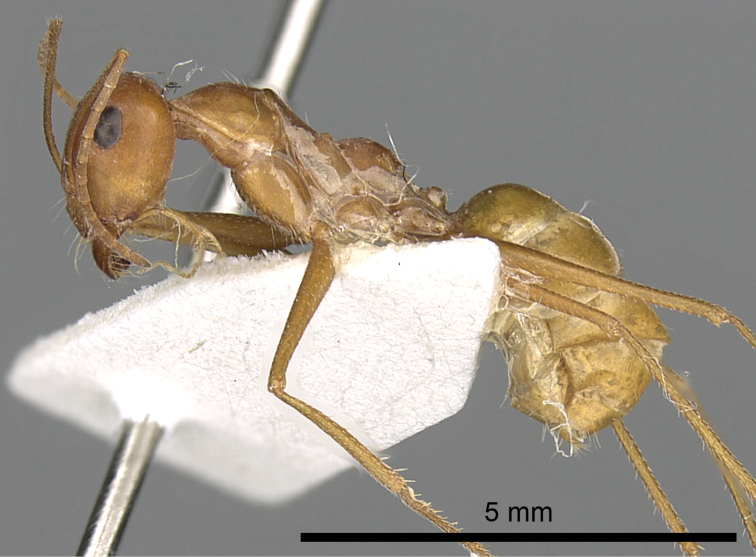
Body in profile of *Cataglyphis flavobrunnea* (paralectotype worker), CASENT0249838.

**Figure 10.**
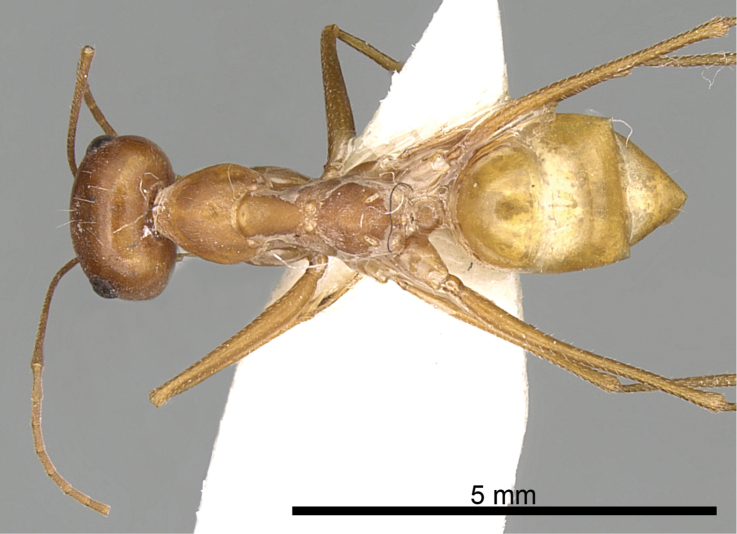
Body in dorsal view of *Cataglyphis flavobrunnea* (paralectotype worker), CASENT0249838.

**Figure 11.**
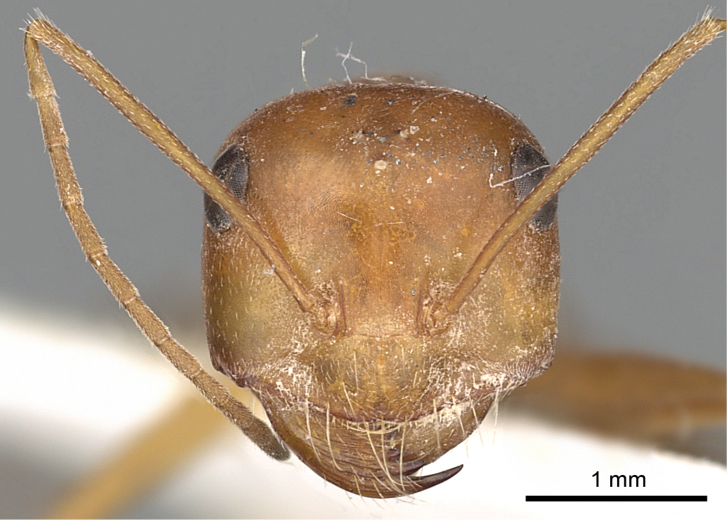
Head in full-face view of *Cataglyphis flavobrunnea* (paralectotype worker), CASENT0249838.

**Figure 12.**
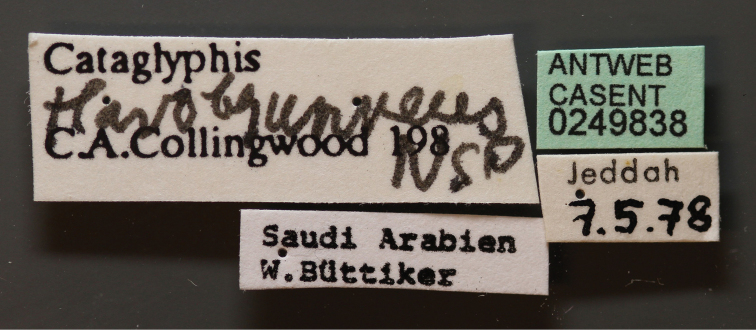
Specimen label. Photographer: Ryan Perry, www.AntWeb.org

Figures 9 , 10 , 11 , 12

Cataglyphis flavobrunneus Collingwood & Agosti, 1996: 378, fig. 41 (w.), Saudi Arabia: Jeddah, 7.v.l978 (W. Buttiker) (NHMB), http://www.antweb.org/specimen/casent0249838 [one paratype worker is designated here as lectotype]. [new designation].

#### Description.

**Major head** HW = HL (CI 100), minor the same with a shallowly convex posterior margin and nearly straight sides; first funicular segment 1.35× longer than second. **Mesosoma.** Metanotal spiracle distinctly raised. **Petiole.** Petiole in profile smoothly rounded but slightly assymetrical with the anterior more rounded and posterior near flat. **Pilosity.** Propodeal dorsum with two pairs of erect hairs; pronotum and mesonotum each with a single pair of erect hairs; posterior margin of head bare; gaster with sparse scattered and very short hairs; all body pubescence whitish or pale. **Sculpture.** Cephalic, mesosomal and petiolar surfaces finely punctate and dull, gaster smooth and shining. **Colour.** Head, mesosoma, petiole, coxae and femora brownish, antennae, tibiae and tarsi yellowish brown, gaster uniform dirty yellow.

#### Measurements.

TL 8.81; HL 1.92; HW 1.82 (major HL = HW = 2.31 Antweb scale); SL 2.21; FS1 0.38; FS2 0.28; PW 1.20; EL 0.53; Indices: CI 95 (Antweb image 100); EI 29; SI 121.

#### Material examined.

Saudi Arabia, Jeddah, 7.5.1978, (W. Buttiker Leg.), 9 workers, major available on http://www.antweb.org/specimen/casent0912239, and minor on http://www.antweb.org/specimen/casent0249839, (NHMB).

#### Remarks.

The original description of *Cataglyphis flavobrunnea* indicated differential diagnosis of this taxon with *Cataglyphis laevior* of the *Cataglyphis bicolor*-group, *diehlii*-complex (Agosti, 1990). However, examination of the newly designated lectotype and the remaining 11 paratypes indicates that the species are very different (casent0104615). *Cataglyphis flavobrunnea* has a uniformly brownish body and yellowish gaster whereas *Cataglyphis laevior* has yellowish brown body and dark brown gaster. The head and mesosoma of *Cataglyphis flavobrunnea* has dense white pubescence, whereas *Cataglyphis laevior* lacks pubescence on the head and mesosoma. Collingwood and Agosti (1996) mentioned that head *Cataglyphis flavobrunnea* is smooth and polished, but the head of the Lectotype is dull and is finely granulate.

### Cataglyphis saharae

Taxon classificationAnimaliaHymenopteraFormicidae

Santschi, 1929

**Figure 13.**
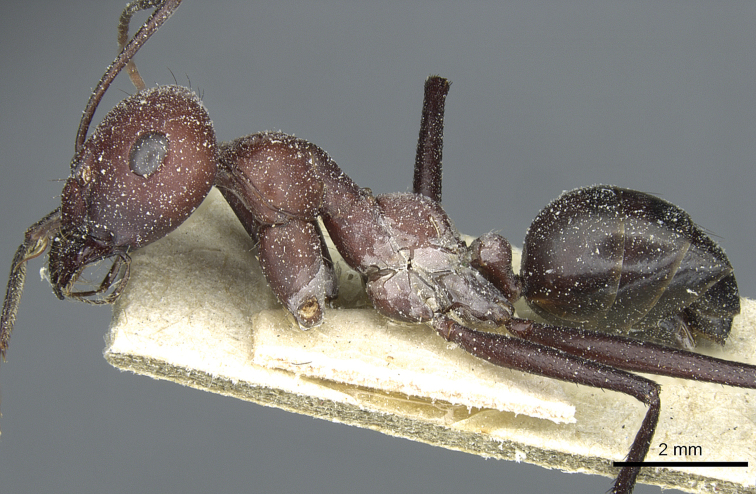
Body in profile of *Cataglyphis saharae* (holotype worker), CASENT0912226.

**Figure 14.**
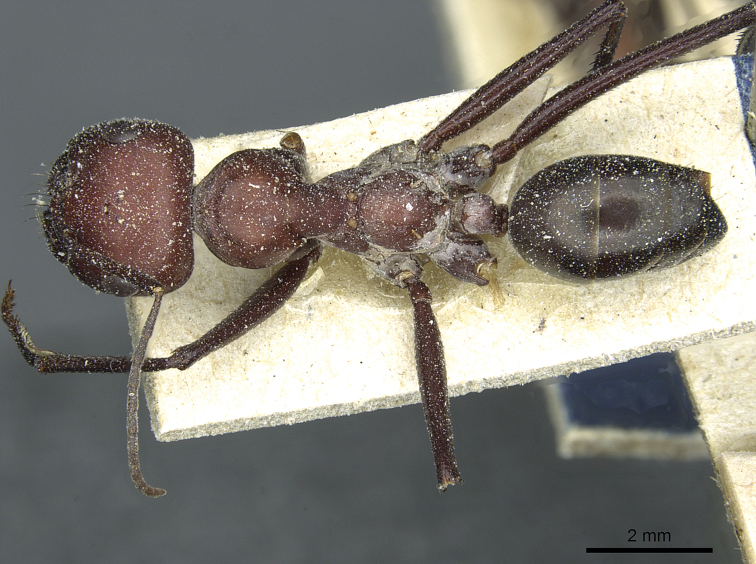
Body in dorsal view of *Cataglyphis saharae* (holotype worker), CASENT0912226.

**Figure 15.**
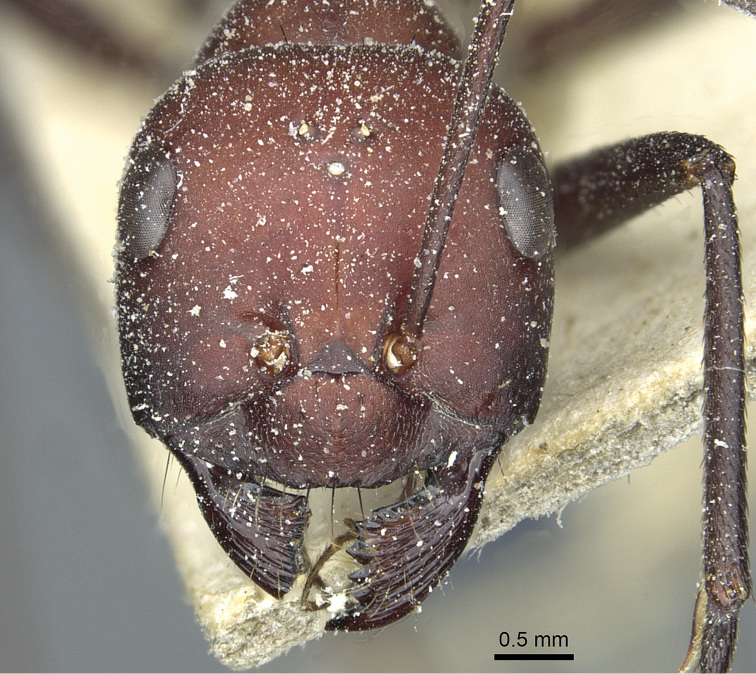
Head in full-face view of *Cataglyphis saharae* (holotype worker), CASENT0912226.

**Figure 16.**
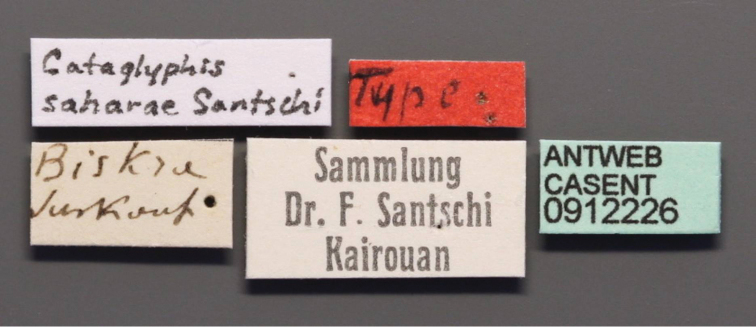
Specimen label. Photographer: Zach Lieberman, www.AntWeb.org

Figures 13 , 14 , 15 , 16

Cataglyphis bicolor st. saharae Santschi, 1929: 48, fig. 3 (w.) (holotype worker), Algeria: Biskra, (NHMB), http://www.antweb.org/specimen/CASENT0912226 [Image of the type specimen examined]. Elevated to species: Collingwood, 1985: 291.
Cataglyphis laylae Collingwood, 2011: 458, pl. 96-103 (w.), United Arab Emirates: Al-Ain [24°13'N 55°46'E], iii.1995, (MHNG), http://www.antweb.org/specimen/CASENT0264538-D01 [holotype presumably lost, paratypes examined], Syn. n.

#### Remarks.

The brief original description did not adequately diagnose this taxon from other members in the *Cataglyphis bicolor*-group. It was mentioned that *Cataglyphis laylae* is similar to *Cataglyphis nigra* (André, 1882) and *Cataglyphis savignyi* (Dufour, 1862) and presented a single distinguishing character, the slender petiole. An examination of several paratypes showed that *Cataglyphis laylae* Collingwood, 2011 is a junior subjective synonym of *Cataglyphis saharae* Santschi.

#### Material examined.

United Arab Emirates, Al-Ain Zoo, 9.v.1995, 24°13'N, 55°46'E, 280 m (C. A. Collingwood leg.) (8 paratype workers) (KSMA), United Arab Emirates, Sweihan, iv.1995 (C. A. Collingwood leg.) (1 paratype worker), United Arab Emirates, Sweihan, iii.1995 (C. A. Collingwood leg.) (5 paratype workers), United Arab Emirates environmental desert, 3.iii.2005 (1), United Arab Emirates, Sharjah desert park, iii. 2006 (1), United Arab Emirates, Al Ain, v. 1995 (Pitfall trap) (1), United Arab Emirates, Sharjah desert, 15.i.2004(2)22 workers with no data, all the materials collected by C. A. Collingwood (WMLC).

## Supplementary Material

XML Treatment for Cataglyphis fisheri

XML Treatment for Cataglyphis flavobrunnea

XML Treatment for Cataglyphis saharae
